# Suicidal risk in patients with aggression in schizophrenia: a systematic review

**DOI:** 10.3389/fpsyt.2025.1560699

**Published:** 2025-04-24

**Authors:** Lidia Bravve, Maria Kaydan, Georgy Kostyuk

**Affiliations:** ^1^ Scientific and Clinical Research Centre for Neuropsychiatry of “Psychiatric Hospital no. 1 Named after N.A. Alexeev of the Department of Health of Moscow, Moscow, Russia; ^2^ Department of Mental Health and Clinical Psychiatry, Faculty of Psychology, Lomonosov Moscow State University, Moscow, Russia; ^3^ Department of Psychiatry and Psychosomatics, Ivan Mikhailovich Sechenov (I. M.) Sechenov First Moscow State Medical University (Sechenov University), Moscow, Russia; ^4^ Department of Psychiatry, Federal State Budgetary Educational Institution of Higher Education Russian Biotechnological University, Moscow, Russia

**Keywords:** schizophrenia, aggression, suicide, suicide risk, aggressive behavior

## Abstract

**Introduction:**

Suicide represents the primary risk factor for mortality among individuals diagnosed with schizophrenia, with a mortality rate that is 10 times higher than that observed in the general population. In the study of individuals who have committed suicide, some exhibited a high risk of aggression and impulsivity, which permitted the consideration of these indicators as predictors of suicide risk. The extant literature contains a number of debates concerning diverse conceptualizations of aggression and impulsivity in the context of suicidal behavior. The present study examined the levels of verbal and physical aggression in individuals diagnosed with schizophrenia, finding that 43% exhibited verbal aggression and 24% physical aggression, levels that are significantly higher than those observed in the general population. Concurrently, an analysis of the psycho-emotional state of patients with suicidal behavior in the anamnesis reveals an indication that the suppression of emotions (including aggression) and the avoidance of harm may result in suicide. This finding is at odds with the previously mentioned results, and consequently, the present review sought to assess the impact of aggressive behavior on suicide risk in patients diagnosed with schizophrenia.

**Methods:**

The search was conducted in accordance with the Preferred Reporting Items for Systematic Reviews and Meta-Analyses (PRISMA) statement. The literature search was conducted in PubMed, Cochrane Library, PsychINFO and Mendeley Data. The search terms used were “aggress*” and “suicid*” or “autoaggress*” and “schizophrenia*”. The search was limited to papers published between 2009 and 2024 (the last 15 years), and the search was continued until November 2024. This systematic review has been registered in PROSPERO (CRD42024628033).

**Results:**

A total of 1,364 articles were identified, 295 of which were duplicates. Following a thorough review, 1,046 articles that did not align with the title and abstract were excluded from the analysis. During the literature search, 23 articles were read in full, of which eight studies met the inclusion criteria. A total of eight studies provided information on the effect of aggressive behavior on suicide in patients with schizophrenia. Of these, six studies demonstrated a positive association between aggression and suicidal behavior or employed a logistic regression model in which the risk of aggression increased the risk of suicide and vice versa. Conversely, two studies revealed no association between suicide risk and aggression.

**Conclusions:**

Patients with a documented history of suicide attempts and aggressive behavior are considered to be at risk of suicide, thus emphasizing the necessity for preventive measures to be implemented for this cohort of patients. However, the conclusion regarding the influence of aggressive behavior on increased suicide risk may not be entirely accurate and may not reflect the true extent of the problem, due to the significant number of methodological inaccuracies and discrepancies in the design of the studies included in the review.

**Systematic review registration:**

https://www.crd.york.ac.uk/PROSPERO/view/CRD42024628033, identifier CRD42024628033.

## 1 Introduction

Suicide represents the primary risk factor for mortality among individuals diagnosed with schizophrenia, with a mortality rate that is 10 times higher than that observed in the general population (1). For instance, the suicide prevalence rate among individuals with schizophrenia is reported to be 352.2 per 100,000 person-years (95% CI 239.3-485.7 per 100,000 person-years) (2), with 44.3% of cases involving a documented suicide plan (3). It has been established that individuals diagnosed with schizophrenia are more likely to employ violent methods in the course of committing suicide, in comparison to the general population. The most prevalent methods include jumping from a height or drowning (4–6). It is estimated that between 50% and 60% of individuals who attempt suicide for the first time will ultimately succumb to their actions (7, 8), the occurrence of a suicide attempt has been demonstrated to result in an elevated risk of subsequent completed suicide (9–11). Risk factors for suicide in schizophrenia include poor compliance with medication, a history of suicide attempts, a sense of hopelessness, male gender, belonging to the white race, tobacco and alcohol use (12), childhood trauma (13–15) and demoralization (16). However, other studies have reported conflicting data on the impact of suicide risk factors such as low education and income, and ethnicity (13). The identification of markers of suicidal behavior in schizophrenia through the study of cognitive function represents a promising area of research. A review study has indicated that a better understanding of the course of the illness among patients, achieved through higher IQ, better executive functioning and higher levels of comprehension, may serve as a predictor of suicidal behavior (17). It is interesting to note that a set of these characteristics offers patients protection against aggressive behavior directed towards others. However, a previous review has already reported deficits in cognitive functions, such as impaired planning and reduced working memory, which contribute to suicidality in schizophrenia (18). The present study suggests that the analysis of a series of cognitive functions may offer a valuable means of identifying individuals who are at risk of suicide. However, the available data are somewhat contradictory. Nevertheless, it is important to consider other risk factors for suicide, as low cognitive functioning is also a risk factor for natural mortality in schizophrenia (19, 20). Moreover, the necessity to ascertain risk factors for suicide is predicated on the objective of preventing it, as there are proven prevention methods for those at high risk, chiefly clozapine (21) and psychotherapeutic methods (22).

Research focusing on individuals who have committed suicide has indicated that some of them exhibit elevated levels of aggression and impulsivity, which can be regarded as indicators of suicide risk (23, 24). There is an ongoing debate in the literature about the different understandings of the terms aggression and impulsivity within the framework of suicidal behavior (25, 26). For instance, impulsivity is defined as “poorly thought out, prematurely expressed, unreasonably risky or inappropriate actions that often lead to undesirable outcomes” (27), whereas aggression is defined as behavior aimed at harming another person motivated to avoid harm (28), expressed verbally and physically (29),Aggression is the sole predictor of suicidal behavior. The prevalence of verbal aggression in individuals diagnosed with schizophrenia is 43%, while the prevalence of physical aggression is 24% (30)), which significantly exceeds the figures observed in the general population (31). The etiology of aggressive behavior in schizophrenia has been linked to structural and functional changes in the brain (32, 33), and alleles of genes associated with an increased risk of aggression have been identified (34, 35). Furthermore, aggression is considered a likely predictor of suicidal behavior, as demonstrated in genetic (36–38), and neuroimaging studies (39–42). The relationship between aggression and suicidal behavior is supported by the fact that the same 5-HT receptors are involved in their pathogenesis: 5-HT1A, 5-HT2A, 5-HT2B, 5-HT2C (43–45). It is evident that individuals diagnosed with schizophrenia who exhibit suicidal tendencies and aggressive behavior constitute a distinct clinical entity. In this group, therapeutic and rehabilitative interventions are likely to be ineffective, as patients with aggressive tendencies often disregard clinical interventions, violate treatment regimens, and evade drug therapy, thereby hindering the efficacy of psychotherapy (46, 47). Conversely, analysis of the psychoemotional state of patients with a history of suicidal behavior reveals evidence that the suppression of emotions (including aggression), and the avoidance of harm, can result in suicide (48–50).

## 2 Methods

### 2.1 Research question

The objective of the present review was to assess the impact of aggressive behavior on suicide risk in patients diagnosed with schizophrenia. We asked to research question:

What is the relationship between suicide risk and aggressive behavior in people with schizophrenia?

### 2.2 Search strategy

This research was conducted in accordance with the Preferred Reporting Items for Systematic Reviews and Meta-Analyses (PRISMA) statement (51). The literature search was conducted by three researchers, who independently and separately searched the three databases: PubMed, Cochrane Library, PsychINFO and in the one secure cloud-based repository Mendeley Data. The search terms used were “aggress*” and “suicid*” or “autoaggress*” and “schizophren*”. The search was limited to works published between 2009 and 2024 (the last 15 years), and the search was continued until November 2024. The complete search terms can be found in **Appendix A**. There were no restrictions on the language of the article. This systematic review was registered with PROSPERO (CRD42024628033).

### 2.3 Inclusion criteria

Inclusion and exclusion criteria are described using the PICOS strategy and displayed in the **Table 1**.

**Table 1:** Eligibility criteria.

	Inclusion	Exclusion
Population	Adult individuals (age, 18–60 years) diagnosed with schizophrenia according to the International Classification of Diseases (ICD-10), DSM-IV, DSM-IV-TR, DSM-IV-R	Children, adolescents, or older adults. Patients with other major disorders, such as bipolar disorder or dementia. Patients with limited physical activity (e.g., cardiopulmonary disease). People with intellectual disabilities.
Intervention	Suicidal risk or suicidal behavior in the history (including completed suicide) and evidence of a relationship between suicidal behavior and aggression	With other mental disorders (depression, borderline personality disorder, and others)
Comparator	Patients with schizophrenia without suicidal risk (thoughts, attempts), or patients with schizophrenia with suicidal risk without aggression, or patients with schizophrenia with aggression without suicidal risk	No comparison group
Outcomes	Identified relationship between aggressive behavior and suicide risk directly using statistical analysis (logistic regression) or objective psychometric measures	No assessment of the relationship between aggressive behavior and suicide risk directly, indirect relationship through other indicators
Study design	Non-randomized studies: case-control, cross-sectional, cohort studies	Systematic reviews, meta-analyses, letter to the editor, abstracts

### 2.4 Data extraction

The selection of research results will be conducted by three independent reviewers (BL, KM, KG), who will utilize the established inclusion/exclusion criteria to make their decisions. In all cases, data were extracted twice, once jointly and once separately by the two authors (BL and KM). The extracted data were then compared, and where discrepancies or disputes were identified, a third author was involved (KG).

Data extraction: the initial phase of this process will be the entry of the data into a standardized spreadsheet by one reviewer. This will be followed by two other reviewers who will then check the extracted data independently. The following data elements were to be extracted: the authors’ names; the country in which the study was conducted; the date of publication; the title of the study; the total sample size; the mean age of the participants; the guidelines according to which a psychiatric diagnosis was made; the principle according to which the research sample was divided into subgroups; the characteristics of the subgroups (number, number of patients with suicidal and separately aggressive behavior); the methods for detecting suicidal and aggressive behavior (medical history data or use of scales); and, in the case of suicide and aggression risk scales, the mean and standard deviation for each group in separate studies.

### 2.5 Methodological quality assessment

The present study examined patients diagnosed with schizophrenia who exhibited either suicidal tendencies or behaviors. The following characteristics were deemed essential in determining the suitability of a study for inclusion: Firstly, patients must be diagnosed with schizophrenia. Secondly, there must be documented history of suicide risk or suicidal behavior, including completed suicide. Thirdly, there must be evidence of an association between suicidal behavior and aggression. Studies were excluded from the review if they did not contain the following information: 1) incorrect description of the sample (missing data for inclusion in the study); 2) information about the study did not match the researchers’ search query. The verification of diagnosis should be conducted in accordance with international clinical guidelines by multiple independent specialists (DSM or ICD criteria could be utilized); the assessment of suicide risk should be performed by a psychiatrist (one or more) according to the following variants: suicidal behavior, presence of suicidal thoughts, completed suicide) or by using psychometric instruments; and the assessment of aggressive behavior, both at the time of the study and of evidence of aggression in the medical history by the following methods: clinical interview or by using psychometric scales; establishment of an association between suicidal behavior and aggression in patients with schizophrenia by correlation or by using logistic regression.

A risk of bias (quality) assessment was conducted.

In accordance with the stipulated guidelines set out in the Cochrane Handbook, the reviewers proceeded to assess the risk of systematic error in each of the included studies. To this end, the ROBINS-E tool was utilized for the purpose of evaluating non-randomized trials. The ROBINS-E tool encompasses the following domains: bias due to confounding, bias due to measurement of exposure, bias in selection of study participants, bias due to post-exposure interventions, bias due to missing data, bias in measurement of outcome, and bias in selection of reported outcome. Each item was then evaluated as being either high risk, causing some concern, or low risk of systematic error. The ROBINS-E (Risk Of Bias In Non-randomized Studies - of Exposures) assessment tool was utilized to evaluate the risk of bias in the study (52). We used the standardized 7 domains to assess risk of bias: 1) risk of bias due to confounding, 2) risk of bias arising from measurement of the exposure, 3) risk of bias in selection of participants into the study (or into the analysis), 4) risk of bias due to post-exposure interventions, 5) risk of bias due to missing data, 6) risk of bias arising from measurement of the outcome, 7) risk of bias in selection of the reported result. Quality assessment was carried out separately by two authors (BL, KM), with a third author involved in the event of disagreement or dispute (KG).

The R package “Robvis” was employed for the purpose of plotting (53).

## 3 Results

### 3.1 Study selection

**Figure 1:**
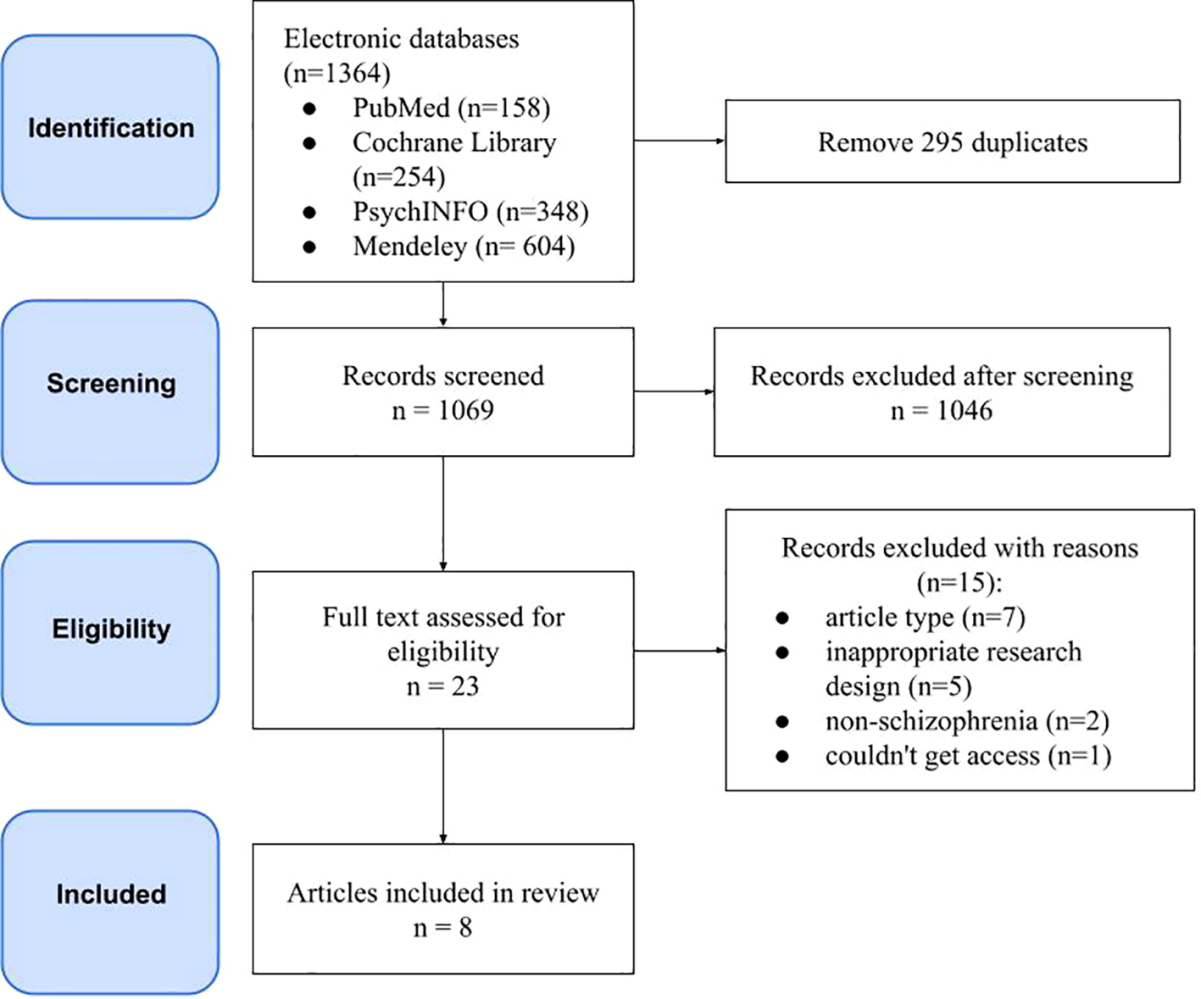
PRISMA flow diagram of systematic review search strategy and study inclusion.

A total of 1,364 articles were identified, 295 of which were duplicates. Following a thorough review, 1,046 articles that did not align with the title and abstract were excluded from further consideration. During the literature search, 23 articles were read in full, of which eight studies met the inclusion criteria. The results of the search and the rationale for exclusion are illustrated in **Figure 1**.

### 3.2 Sample characteristics

A total of eight studies were included in the analysis, providing data on 102,760 patients diagnosed with mental disorders, of whom 10,698 had been diagnosed with schizophrenia (54–61). Patients were recruited from hospitals in China, Canada, Germany, Turkey, Norway, Israel and France. The mean age of the study participants was reported in 5 articles (55, 57, 58, 60, 61) and was 37.5 ± 11.9, this indicator was not provided by the other studies (54, 56, 59). The total number of cases of schizophrenia and suicide in all eight studies was 624. In one article, however, the exact number of patients with suicidal behavior was difficult to calculate because the main group was considered to be patients with schizophrenia with only “inpatient suicide” and “suicide attempt (admission)” (n=47), which were included in the total number of patients with schizophrenia and suicide (60). The total number of patients with aggressive behavior was extracted from four articles, yielding a total of 1,294 patients (54, 56, 59, 61). In three articles, the calculation of the number of aggressive patients was not possible due to the provision of only parametric indicators of the aggressiveness of the groups (55, 57, 58). In one study, the precise number of patients with aggressive behavior could not be ascertained, as it is possible for the same patients to be included in different groups, e.g. ‘physical assault admission’, ‘physical assault inpatient’, ‘threatening behavior admission’ or ‘damage to property admission’ (60). In one study, the precise number of patients with aggressive behavior could not be ascertained due to the inclusion of the same patients in different groups, e.g. ‘lifetime suicide attempt’, ‘inpatient suicide attempt or suicidal ideation’ and ‘admission for physical assault’, ‘admission for physical assault inpatient’, ‘admission for threatening behavior’ or ‘admission for damage to property’ (60). Three studies reported the number of patients with schizophrenia who exhibited suicidal behavior and aggression, constituting 62% (n=133) of all patients with schizophrenia and suicide included in these studies (54, 56, 59).

### 3.3 Suicide assessment

Three studies utilized psychometric scales to evaluate suicide risk upon admission to hospital: Suicide Risk Assessment (SRS) (54, 57), Nurses’ Assessment of Suicide Risk for Suicide (54), and The InterSePT Scale for Suicidal Thinking (ISST) (55). In three studies, suicidal behavior was assessed according to the following criteria: history of lifetime suicide attempts (58), suicidal behavior in hospital including completed suicide (60), and completed suicide only (59). However, it should be noted that in two studies, the circumstances and time of occurrence of suicidal thoughts and attempts remain unreported (56, 61).

### 3.4 Aggressive assessment

In the five articles, the following psychometric scales were utilized to evaluate the level of aggression at hospital admission: two articles employed the Overt Aggression Scale (OAS) (57, 58), in one study the Modified Overt Aggression Scale (54) was used, in another the Buss-Perry Aggression Questionnaire (BPAQ) (56) was employed, and in the last study a separate item on the scale was taken into account: Positive and Negative Syndrome Scale “Disordered regulation and control of action on inner urges/emotions” (G14) (55). In the other two articles, the assessment of aggressive behavior was conducted in accordance with two distinct methodologies. Firstly, the occurrence of aggression was gauged by examining episodes of aggression within the hospital setting and the individual’s medical history (60). Secondly, the assessment of aggressive behavior was undertaken 12 months prior to the occurrence of suicide (59). It is noteworthy that one study did not report the circumstances of the occurrence of aggression (61).

### 3.5 Design of the research and diagnosis verification

The extant literature on this subject comprises two case-control studies (54, 59), five cross-sectional studies (55–57, 60, 61), and one cohort study (58). The diagnoses were verified using the following diagnostic systems: ICD-10 (54, 60), DSM-IV (55, 56, 59, 61), DSM-IV-TR (57), DSM-IV-R (58). Patients were examined by a psychiatrist and a psychologist (56, 58), a specialist and a psychiatric assistant (61), and two psychiatrists (57). The number of specialists (psychiatrists and psychologists) who confirmed the diagnosis, collected the anamnestic data and assessed the psychometric indicators was not indicated in (54, 55, 59, 60).

### 3.6 Studies on aggression and suicide in schizophrenia patients

A total of eight studies incorporated data pertaining to the impact of aggressive behavior on suicide in patients diagnosed with schizophrenia (see **Table 2**). In the study (56), aggressive behavior was found to be associated with suicidal behavior in 50% of patients with aggression (χ 2 = 6,276, p=0,012). Furthermore, a higher mean score on the OAS scale was observed in patients with a history of suicide attempts (58) when compared to those without (3 versus 1.8, p = 0.03). The number of previous suicide attempts exhibited a positive correlation with the score on the OAS scale (p = 0.001). The study (59) included patients diagnosed with schizophrenia who had completed suicide and whose history was studied retrospectively. The study found an association between suicide and aggressive behavior in 38% of cases (χ 2 = 13.16, p=0.0003).

**Table 2:** Study characteristics and main findings of included studies (N=8).

Author, year (country)	Sample (n)	Diagnostic classification	Included diagnosis	Age (mean SD)	Research groups (n)	Suicidal behavior/attempts	Aggression	Suicide assessment tool	Aggression assessment tool	Scale score	The relationship between aggression and suicide risk
Lin Y. et al., 2023 (54)(China)	849 (schizophrenia n = 523)	ICD-10	Schizophrenia, bipolar disorder, depression, others	–	1) Suicidal intention (n=270, with schizophrenia = 157), 2) Non-Suicidal intention (n=579, with schizophrenia = 366)	Psychometric scale	Psychometric scale	Suicide Risk Assessment Scale, Nurses’ Assessment of Suicide Risk for Suicide	Modified Overt Aggression Scale	–	Overt aggression linked to suicidal behavior
Tousignant M. et al.,2010 (59) (Canada)	67	DSM-IV	Schizophrenia	–	1) Suicide with schizophrenia - n=33, 2) Control group (schizophrenia without suicide) - n= 34	Anamnestic data	Anamnestic data	–	–	–	Suicide was associated with aggressive behavior
Neuner T. et al., 2011 (60) (Germany)	49257 (schizophrenia n= 8901)	ICD-10	Schizophrenia and other disorders	40,4 ± 13,6	1) Suicidal behavior/attemps with schizophrenia - n=47, 2) Control group (other mental illness with suicidal behavior/attemps) - n= 216	Anamnestic data	Anamnestic data	–	–	–	Suicidal behavior or attempts were not associated with a history of violent behavior
Köşger F. et. al., 2016 (56) (Turkey)	68	DSM-IV	Schizophrenia	–	1) Aggressive behavior in patients with schizophrenia - n=30 (with suicide = 15), 2) No aggressive behavior in patients with schizophrenia - n=38 (with suicide = 8)	Anamnestic data	Psychometric scale	–	Buss–Perry Aggression Questionnaire (BPAQ)	BPAQ group with aggressive behavior - physical aggressive = 17.07 ± 11.18, verbal = 9.50 ± 4.46	Aggressive behavior was associated with suicidal behavior
Mork E. et. al., 2013 (55) (Norway)	251	DSM-IV	Schizophrenia	30,1 ± 9,8	1) Suicide attempt and self-harm (SA + NSSH) - n=36, 2) Only suicide attempt (SA only) - n=52, 3) No suicide attempt and self-harm (NoSA) - n=163	Psychometric scale	Psychometric scale	The InterSePT Scale for Suicidal Thinking (ISST)	Positive and Negative Syndrome Scale (PANSS) G14 (Disordered regulation and control of action on inner urges/emotions)	ISST - No current suicidality (0), n (%): SA + NSSH = 13 (36); SA only = 27 (53); NoSA = 117 (72); Low suicidality (0.1-1.0), n (%): SA + NSSH = 14 (39); SA only = 20 (39); NoSA = 41 (25); Moderate to high (1.1-2.0), n (%): SA + NSSH = 2 (6); SA only =6 (12); NoSA = 8 (5); PANSS G14 (median (min-max)): SA + NSSH = 2 (1-6); SA only = 1 (1-4); NoSA = 1 (1-4)	Group pf Suicide attempt and self-harm had higher scores on current impulsive aggression and depressive symptoms
Iancu I. et al., 2010 (57) ()(Israel, USA)	68	DSM-IV-TR	Schizophrenia	39,4 ± 12,17	1) Low impulsivity (n=35) (with suicide = 9), 2) high impulsivity (n=33) (with suicide = 15)	Psychometric scale	Psychometric scale	Suicide Risk Scale (SRS)	Overt Aggression Scale (OAS)	OAS for low impulsivity group = 5.5183.40, for high impulsivity group = 7.2483.61; SRS for low impulsivity group = 8.7784.49, for high impulsivity group = 11.6484.24	Older age and higher rates of aggression, impulsivity and general psychopathology on the PANSS subscale had significant contribution to the explained variance in predicting suicide risk
Lejoyeux M. et al., 2013 (58)(France)	100	DSM-IV-R	Schizophrenia	41,9 ± 13,1	The authors of the study did not divide the patients into subgroups. However, an analysis of the baseline data presented in the study did allow for the identification of suicidal behavior in the sample. The following division of the sample was revealed: 1) Suicide with schizophrenia - n=53, 2)Control group (schizophrenia without suicide) - n= 47	Anamnestic data	Psychometric scale	–	Overt Aggression Scale (OAS)	OAS with suicide group 3 ± 3, without suicide = 1.8 ± 2.4	Patients with a history of suicide attempts had higher scores on the Overt Aggression Scale (OAS)
Yıldız M., 2010 (61)(Turkey)	720	DSM-IV	Schizophrenia	35,5 ± 11,0	Suicide with schizophrenia - n=199, second group (schizophrenia without suicide) = 521; aggression with schizophrenia = 341, second group (schizophrenia without aggression) = 379	Anamnestic data	Anamnestic data	–	–	–	The presence of physically aggressive behavior is associated with suicidal thoughts and suicidal attempts

The remaining studies used logical regression to analyses the association between different factors (including aggression and suicidal behavior) (54) found an association between overt aggression and some other sociodemographic and clinical-dynamic factors with the risk of suicidal behavior in patients with severe mental illness (including schizophrenia) (OR = 2.008, 95% CI: 1.410, 2.861) (55) also included cases of self-harm without the intention to die in the group with suicidal behavior. Patients with suicidal behavior and self-harm without intent to die had higher scores for current impulsive aggression and depressive symptoms than patients with schizophrenia without suicidal behavior, but statistical analysis did not reveal significant differences (57) developed a predictive model and found that older age and higher rates of aggression, impulsivity and general psychopathology on the PANSS subscale made a positive and significant contribution to the explained variance in predicting suicide risk (Exp(B) = 0.67, β = 0.36, p < 0.001) (61) also predicted suicide risk. The presence of physically aggressive behavior has been found to be associated with the following variables: suicidal thoughts and attempts (χ2 = 33324, p = 0.001), suicide attempts (χ2 = 8253, p = 0.004), and a criminal record (χ2 = 22410, p = 0.001). The findings indicate that suicidal thoughts and attempts significantly elevate the risk of physically aggressive behavior, with a reported threefold increase (β = 3.17) (60). However, a separate report states that suicidal behavior or attempts are not associated with a history of violent behavior (OR 4.584, p = 0.145). However, difficulties arose during the interpretation of results due to the fact that the primary group with suicide in schizophrenia included only patients who had committed or attempted suicide in hospital. The logistic regression was carried out on 5241 patients, taking into account the suicide sign on admission to hospital, which was indicated for a larger number of patients than in the study group (60).

### 3.7 Quality assessment

**Figure 2:**
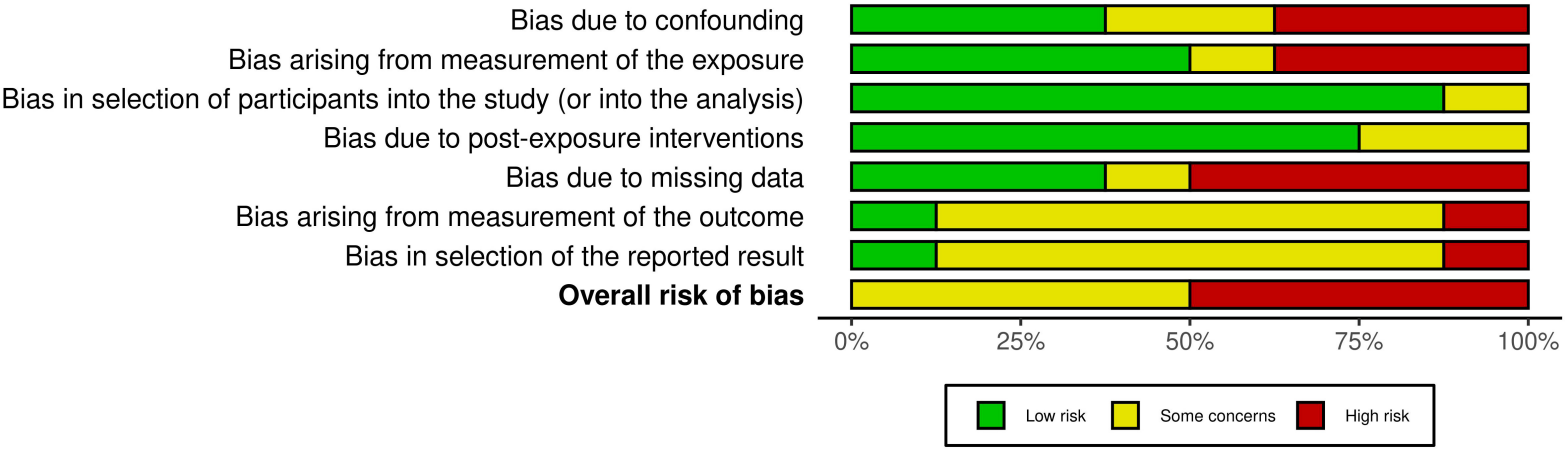
ROBINS-E tool for non-randomized controlled trials.

**Figure 3:**
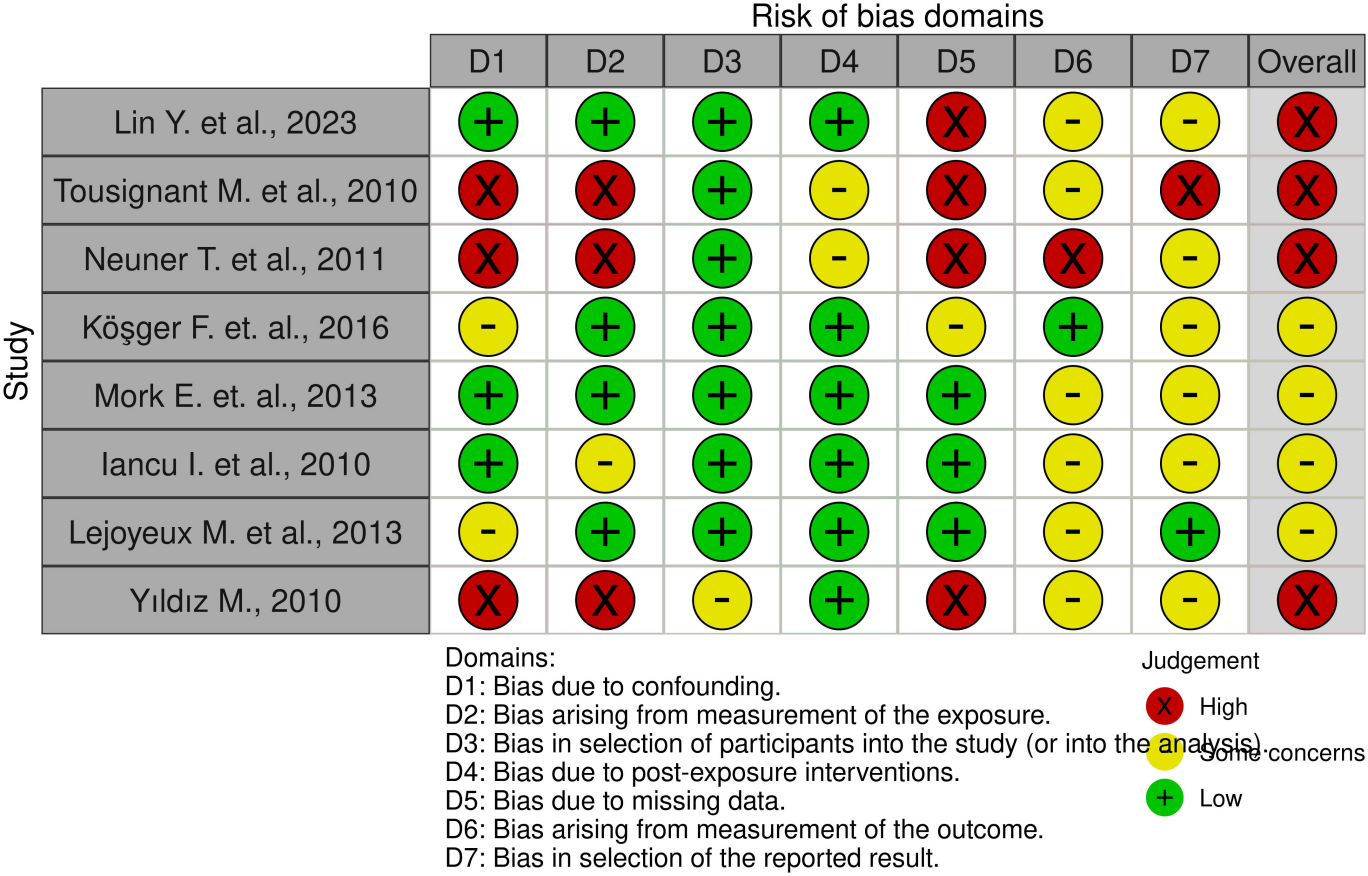
ROBINS-E tool for non-randomized controlled trials (summary graph).

The results are demonstrated in **Figures 2** and **3**.

The quality assessment identified 4 studies with a high risk of bias and 4 studies with some concern about bias. The high risk was primarily attributable to ambiguity in the measurement of exposure or an absence of data. For instance, in the study conducted by Tousignant et al. (2011), it was not possible to ascertain the severity of indicators of aggression and suicidal risk in relation to the completed suicide of the primary group (data on the presence of aggressive behavior, suicide attempts in the history were obtained from the words of relatives) (60). The absence of psychometric averages and the presence of gaps in the analyzed data are notable. The analysis was conducted on a different number of subjects, and the data lacked psychometric indicators and separation into groups. However, it did indicate the number of patients with schizophrenia who had both suicidal attempts and aggressive behavior. The study (54) demonstrates an absence of average indicators in terms of socio-demographic, clinical, dynamic and psychometric indicators. Concurrently, the presence of comorbid disorders (e.g., alcoholism, surfactant dependence) was not consistently considered, potentially introducing a high risk of bias. In the remaining articles, concerns regarding risk of bias were identified, stemming from the inability to assess the researcher’s bias in measuring outcomes, particularly in the context of data presented in medical records and the collection of anamnestic data from relatives. The principal outcome of the risk of bias assessment was an understanding of the impossibility of performing a meta-analysis for the studies selected in the systematic review.

## 4 Discussion

This review presents evidence on the association between suicide risk and aggressive behavior in patients diagnosed with schizophrenia. The majority of studies indicate a correlation between aggression and suicidal behavior (55–59, 61), with this relationship manifesting in various forms. A study of the history of patients who have committed suicide (59) demonstrated that suicidal behavior is associated with elevated levels of mental vulnerability, including aggressive behavior. When discussing aggressive behavior in schizophrenia, it is imperative to address its comorbidity with other psychiatric disorders, as evidenced by the fact that up to 47% of patients also suffer from concomitant substance dependence (46), which in itself increases the risk of aggression (62, 63). Furthermore, it is imperative to acknowledge that depression, a common comorbidity in schizophrenia, is present in 50% of cases (64), and has been demonstrated to heighten the risk of suicidal behavior (65, 66). Furthermore, a study (58) revealed that patients with a history of suicidal behavior exhibited higher levels of aggression upon admission to hospital (56) found that aggressive patients had a greater history of suicide attempts than patients without aggression. These results are consistent with those of previous studies, which found that patients with suicidal behavior in schizophrenia had increased levels of impulsive-aggressive traits compared to patients with suicidal behavior in other psychiatric disorders (67). However, there are some conflicting studies. For example, a paper (68) like the one we included (55), examined risk factors for suicidal behavior in schizophrenia by measuring the PANSS scale item ‘disordered regulation and control of action on inner urges/emotions’ (G14). This item was not considered to be a reflection of aggression, and no significant differences were found between schizophrenic patients with suicidal and non-suicidal behavior. In a further noteworthy study (69), a relationship was identified between empathy and neurocognitive functions and the development of suicidal thoughts in schizophrenia among Chinese patients. The authors demonstrated that the general psychopathological symptoms of the PANSS scale were associated with the occurrence of suicidal ideation. However, no separate comparison was provided for the subscale items (69). Furthermore, an increased propensity for aggression has been documented in patients who have attempted suicide in the context of major depressive disorder (70). Additionally, patients with suicidal ideation in depression and bipolar disorder have exhibited higher levels of impulsivity and hostility compared to those without such ideation (71). However, these findings appear to be at odds with the established link between suicidal thoughts and higher harm avoidance (72), suggesting that any act of aggression towards others poses a threat to the aggressor. However, it is important to note that in schizophrenia, there are pronounced cognitive and sensory processing impairments (73, 74), which can result in an underestimation of the stressful situation and a potential lack of understanding of the consequences of one’s behavior. Consequently, patients may have an impaired understanding of self-harm.

In other studies incorporated within the scope of this review, the correlation between suicide risk and aggressive behavior is demonstrated through the utilization of mathematical models, namely the construction of predictive models. Aggression was identified as one of several factors that augment the risk of suicide. The utilization of mathematical models facilitates the streamlining and standardization of the identification of patients at risk of suicide. For instance (54, 57), predicted suicide risk by noting that aggression was one of the risk factors, while (61) predicted aggressive behavior in schizophrenia and found that suicidal thoughts and attempts increased it. It is noteworthy that analogous predictive models have been constructed for other disorders, though the factor of aggression was not identified among them. A notable study predicted the overlap between suicide attempt and aggression in young adults, concluding that age and education level were significant risk factors (75). In predicting suicide risk subsequent to a suicide attempt, variables such as psychotic disorders and depression have been identified as significant factors (76). Moreover, data from a meta-analysis demonstrated that experiencing psychosis increased the risk of suicidal ideation (77). Conversely, significant factors in predicting suicide attempt in major depressive disorder were comorbid personality disorder (78) and previous suicidal behavior (79), occurrence of suicidal ideation within a brief period following the initial depressive episode, lower secondary education and comorbid psychiatric disorder (80). Predictors of making a first suicide attempt in depression are high anxiety, elevated cholesterol and thyroid-stimulating hormone levels (81), and in bipolar disorder, early onset, type I disorder, comorbidity and some neuroimaging parameters (82). Consequently, the integration of predictive methods into contemporary clinical practice holds considerable promise for enhancing diagnostic accuracy (83, 84). However, it is important to note that the widespread implementation of these methods is constrained by a number of limitations (85, 86), primarily due to the multifaceted nature of factors identified as suicide predictors (87).

Concurrently, certain studies have demonstrated an absence of correlation between suicidal risk and aggression. In the studies referenced as (60) and (55), higher psychometric measures of aggression were observed in the group of patients with suicidal behavior and self-harm without intent to die, in comparison to patients with schizophrenia without suicide. However, these results did not reach significant values. A number of studies addressing diverse mental illnesses have not directly considered the aggression factor, yet have demonstrated the significance of violent episodes (88, 89). Conversely, other works have been published that do not consider aggression as a marker of suicide risk in schizophrenia, and therefore it has not been measured with psychometric tools or taken into account in the history (90–92).

In conclusion, it is imperative to acknowledge that patients who have attempted suicide constitute a high-risk group for making subsequent attempts within the initial year following hospital discharge. This risk persists even after the completion of their treatment (93–95). Consequently, patients exhibiting suicidal tendencies and a documented history of aggressive behavior necessitate expeditious and comprehensive psychiatric care, entailing the prescription of ant suicidal medications (21), the implementation of strategies to mitigate aggression (96–98), and the provision of efficacious psychotherapy techniques (99). It is important to note that patients with a history of aggressive behavior are less likely to adhere to therapy, which can complicate the treatment process (100–102). The implementation of preventive measures aimed at averting aggressive behavior in patients diagnosed with schizophrenia is associated with a number of limitations. For instance, risk factors for the development of aggressive behavior in adulthood may include physical and emotional abuse during childhood, which can only be assessed retrospectively (103, 104). This underscores the necessity for preventative interventions by risk groups well in advance of the onset of aggressive symptoms (dysfunctional families). The study of genetic markers (105) and environmental factors (106–109) is a promising avenue for further research.

Another promising direction is treatment aimed at improving cognitive functioning, as a number of cognitive functions are altered in schizophrenia with aggressive behavior: for example, worse working memory but better attention has been reported (110, 111). Some research links cognitive impairment to suicide (112, 113), others say there’s no change (17, 114). It is also important to recognize that cognitive decline is one of the axial symptoms of schizophrenia (115–117)., therefore a search for more reliable markers is required. As such, cognitive functioning is limited to be used as a major point of application in therapy. Despite this, it is necessary to study cognitive functioning, which is clearly altered in aggression and suicidal behavior in schizophrenia, as there are ways to correct them. Commonly recognized methods with proven efficacy are the administration of second-generation antipsychotics (118, 119), work with a psychotherapist (110, 120), range of physical activities (121), non-invasive brain stimulation techniques (122). Once again, we note that we cannot speak of the specificity of cognitive impairment, as it is one of the persistent symptoms of schizophrenia that determines mortality in this cohort of patients (19, 20). This area of research is of interest in terms of preventive interventions, as a number of studies have shown that some cognitive abilities are innate (123, 124) or appear earlier than the rest of the symptoms of schizophrenia (125–127). If specific changes in aggression and suicide can be identified in schizophrenia, it will help to identify a group at risk before a suicide attempt or suicidal ideation occurs.

A subsequent analysis of the design and methodology of the included studies identified several limitations. For instance, despite the unifying subject of the single study, there were considerable variations in the methods employed to obtain patient information and psychometric tools, which rendered the analysis of the results challenging. For instance, the article (60) conceptualized physical violence and property damage as manifestations of aggression, the paper (59) documented death threats or assault, the third article exclusively measured physical aggression (61), while the remaining articles employed scales to assess admission to hospital. A similar discrepancy was observed in the case of suicidal behavior assessment in the aforementioned articles. Returning to the methodology of the study, the question arises as to whether it is legitimate to include works and studies on completed suicide and on suicide attempts together in the observation. For instance, the review included a case-control study where the clinical sample comprised cases of completed suicide (59), yet there remains a paucity of understanding regarding whether the disorders identified in suicide autopsies are indicative of individuals who have made suicide attempts (128–130). In one study, the primary sample comprised patients with various psychiatric disorders in addition to schizophrenia, which limits the generalizability of the findings (54). Furthermore, the categorization of study groups is inconsistent, with the majority divided according to the presence or absence of suicidal behavior. However, two studies utilized aggressive (56) or impulsive (57) behavior as the basis for their group divisions. A further noteworthy point pertains to the heterogeneity of the comparison groups. In two studies, the control groups comprised other diagnoses in addition to schizophrenia, thereby introducing further complexity in the analysis of the data (54, 60).

It is important to note that the majority of the studies reviewed did not include substance and nicotine dependence as exclusion criteria. However, it is well-established that substance dependence is a proven risk factor for aggressive behavior (131–133). For instance, the inclusion of patients diagnosed with schizophrenia who also consumed psychoactive substances resulted in distorted outcomes. A mere two studies incorporated substance dependence as an exclusion criterion (54, 56), with the findings of one study being constrained by the fact that the study group comprised patients diagnosed with schizophrenia and additional psychiatric disorders, as previously discussed. In the second study, the basis for group segmentation was not based on patients’ suicidal behavior but rather on their aggressive behavior.

## 5 Conclusion

In summary, the conclusion regarding the impact of aggressive behavior on elevated suicide risk may be deemed as somewhat erroneous. This is due to the significant number of methodological inaccuracies and inconsistencies in the study design that were identified in the included reviews. Consequently, this may not accurately reflect the actual state of the problem.

## Data Availability

The original contributions presented in the study are included in the article/supplementary material, further inquiries can be directed to the corresponding author.

## Appendix A

**Search strategy for the PubMed database:**

Search: (((aggress*) AND (suicid*)) OR (autoaggress*)) AND (schizophren*) Filters: from 2009 - 2024

**Search strategy for the Cochrane Library:**

(aggress*):ti,ab,kw AND (suicid*):ti,ab,kw OR (autoaggress*):ti,ab,kw AND (schizophren*):ti,ab,kw with Cochrane Library publication date Between Jan 2009 and Jan 2024 (Word variations have been searched)

**Search strategy for the PsychINFO:**

it was carried out by several consecutive queries in accordance with the key search words in previous cases with a limit of one operator: aggress* AND suicid* OR autoaggress* AND schizophren*

**Search strategy for the Mendeley:**

Two searches were performed in succession and the results were included in the study:

aggress suicid schizophren Between 2009 and 2024

aggress autoaggress schizophren Between 2009 and 2024

## References

[1] Correll CU Solmi M Croatto G Schneider LK Rohani-Montez SC Fairley L . Mortality in people with schizophrenia: a systematic review and meta-analysis of relative risk and aggravating or attenuating factors. World Psychiatry. (2022) 21:248–71. doi: 10.1002/wps.20994 PMC9077617 35524619

[2] Fu XL Qian Y Jin XH Yu HR Wu H Du L . Suicide rates among people with serious mental illness: a systematic review and meta-analysis. Psychol Med. (2023) 53:351–61. doi: 10.1017/S0033291721001549 33952359

[3] Bai W Liu ZH Jiang YY Zhang QE Rao WW Cheung T . Worldwide prevalence of suicidal ideation and suicide plan among people with schizophrenia: a meta-analysis and systematic review of epidemiological surveys. Transl Psychiatry. (2021) 11:552. doi: 10.1038/s41398-021-01671-6 34716297 PMC8556328

[4] Hunt IM Kapur N Windfuhr K Robinson J Bickley H Flynn S . Suicide in schizophrenia: findings from a national clinical survey. J Psychiatr Pract. (2006) 12:139–47. doi: 10.1097/00131746-200605000-00002 16732132

[5] Pan CH Chen PH Chang HM Wang IS Chen YL Su SS . Incidence and method of suicide mortality in patients with schizophrenia: a Nationwide Cohort Study. Soc Psychiatry Psychiatr Epidemiol. (2021) 56:1437–46. doi: 10.1007/s00127-020-01985-8 33245380

[6] Trott M Suetani S Arnautovska U Kisely S Kar Ray M Theodoros T . Suicide methods and severe mental illness: A systematic review and meta-analysis. Acta Psychiatr Scand. (2024) 151(4):467–84. doi: 10.1111/acps.13759 PMC11884913 39350700

[7] Isometsä ET Lönnqvist JK . Suicide attempts preceding completed suicide. Br J Psychiatry. (1998) 173:531–5. doi: 10.1192/bjp.173.6.531 9926085

[8] Yook V Kim H Kim EJ Kim Y Lee G Choi JH . Psychological autopsy study comparing suicide decedents with and without a history of suicide attempts in a nationwide sample of South Korea. Suicide Life Threat Behav. (2022) 52:190–8. doi: 10.1111/sltb.12750 33811661

[9] Goñi-Sarriés A Blanco M Azcárate L Peinado R López-Goñi JJ . Are previous suicide attempts a risk factor for completed suicide? Psicothema. (2018) 30:33–8. doi: 10.7334/psicothema2016.318 29363468

[10] Bostwick JM Pabbati C Geske JR McKean AJ . Suicide attempt as a risk factor for completed suicide: even more lethal than we knew. Am J Psychiatry. (2016) 173:1094–100. doi: 10.1176/appi.ajp.2016.15070854 PMC5510596 27523496

[11] Suominen K Isometsä E Suokas J Haukka J Achte K Lönnqvist J . Completed suicide after a suicide attempt: a 37-year follow-up study. Am J Psychiatry. (2004) 161:562–3. doi: 10.1176/appi.ajp.161.3.562 14992984

[12] Cassidy RM Yang F Kapczinski F Passos IC . Risk factors for suicidality in patients with schizophrenia: A systematic review, meta-analysis, and meta-regression of 96 studies. Schizophr Bull. (2018) 44:787–97. doi: 10.1093/schbul/sbx131 PMC6007264 29036388

[13] Benster LL Stapper N Rodriguez K Daniels H Villodas M Weissman CR . Developmental predictors of suicidality in schizophrenia: A systematic review. Brain Sci. (2024) 14(10):995. doi: 10.3390/brainsci14100995 39452009 PMC11506348

[14] Hassan AN Stuart EA De Luca V . Childhood maltreatment increases the risk of suicide attempt in schizophrenia. Schizophr Res. (2016) 176:572–7. doi: 10.1016/j.schres.2016.05.012 27236409

[15] Alli S Tasmim S Adanty C Graff A Strauss J Zai C . Childhood trauma predicts multiple, high lethality suicide attempts in patients with schizophrenia. Psychiatry Res. (2019) 281:112567. doi: 10.1016/j.psychres.2019.112567 31586840

[16] Berardelli I Sarubbi S Rogante E Hawkins M Cocco G Erbuto D . The role of demoralization and hopelessness in suicide risk in schizophrenia: A review of the literature. Medicina. (2019) 55:200. doi: 10.3390/medicina55050200 31126145 PMC6571661

[17] Tomé-Fernández M Berbegal-Bernabeu M Sánchez-Sansegundo M Zaragoza-Martí A Rubio-Aparicio M Portilla-Tamarit I . Neurocognitive suicide and homicide markers in patients with schizophrenia spectrum disorders: A systematic review. Behav Sci (Basel). (2023) 13(6):446. doi: 10.3390/bs13060446 37366698 PMC10295345

[18] Le GH Wong S Haikazian S Johnson DE Badulescu S Kwan ATH . Association between cognitive functioning, suicidal ideation and suicide attempts in major depressive disorder, bipolar disorder, schizophrenia and related disorders: A systematic review and meta-analysis. J Affect Disord. (2024) 365:381–99. doi: 10.1016/j.jad.2024.08.057 39168166

[19] Dickerson F Khan S Origoni A Rowe K Katsafanas E Harvin A . Risk factors for natural cause mortality in schizophrenia. JAMA Netw Open. (2024) 7:e2432401. doi: 10.1001/jamanetworkopen.2024.32401 39254976 PMC11388031

[20] Mohn C Olsson AK van Dijk Härd I Helldin L . Neurocognitive function and mortality in patients with schizophrenia spectrum disorders. Schizophr Res Cognit. (2023) 33:100284. doi: 10.1016/j.scog.2023.100284 37078076 PMC10106500

[21] Masdrakis VG Baldwin DS . Prevention of suicide by clozapine in mental disorders: systematic review. Eur Neuropsychopharmacol. (2023) 69:4–23. doi: 10.1016/j.euroneuro.2022.12.011 36640481

[22] Bornheimer LA Verdugo JL Brdar NM Im V Jeffers N Bushnell CB . A cognitive-behavioral treatment for suicide prevention among adults with schizophrenia spectrum disorders in community mental health: Study protocol for a pilot feasibility and acceptability randomized clinical trial. Pilot Feasibility Stud. (2024) 10:99. doi: 10.1186/s40814-024-01523-2 38997747 PMC11241875

[23] Gvion Y Levi-Belz Y . Serious suicide attempts: systematic review of psychological risk factors. Front Psychiatry. (2018) 9:56. doi: 10.3389/fpsyt.2018.00056 29563886 PMC5845877

[24] Levi-Belz Y Gvion Y Apter A . The serious suicide attempts approach for understanding suicide: review of the psychological evidence. Omega (Westport). (2022) 86:591–608. doi: 10.1177/0030222820981235 33327864

[25] Gvion Y Apter A . Aggression, impulsivity, and suicide behavior: A review of the literature. Arch Suicide Res. (2011) 15(2):93–112. doi: 10.1080/13811118.2011.565265 21541857

[26] Moore FR Doughty H Neumann T McClelland H Allott C O’Connor RC . Impulsivity, aggression, and suicidality relationship in adults: A systematic review and meta-analysis. EClinicalMedicine. (2022) 45:101307. doi: 10.1016/j.eclinm.2022.101307 35243273 PMC8860929

[27] Evenden JL . Varieties of impulsivity. Psychopharmacol (Berl). (1999) 146:348–61. doi: 10.1007/pl00005481 10550486

[28] Baron RA Richardson D . Human aggression. New York, NY: Plenum Press. 1994. 308 p. Available at: https://play.google.com/store/books/details?id=bZK1BwAAQBAJ (Accessed January 04, 2025).

[29] Buss AH Perry M . The aggression questionnaire. J Pers Soc Psychol. (1992) 63:452–9. doi: 10.1037//0022-3514.63.3.452 1403624

[30] Li W Yang Y Hong L An FR Ungvari GS Ng CH . Prevalence of aggression in patients with schizophrenia: A systematic review and meta-analysis of observational studies. Asian J Psychiatr. (2020) 47:101846. doi: 10.1016/j.ajp.2019.101846 31715468

[31] Witt K van Dorn R Fazel S . Risk factors for violence in psychosis: systematic review and meta-regression analysis of 110 studies. PLoS One. (2013) 8:e55942. doi: 10.1371/journal.pone.0055942 23418482 PMC3572179

[32] Wong TY Radua J Pomarol-Clotet E Salvador R Albajes-Eizagirre A Solanes A . An overlapping pattern of cerebral cortical thinning is associated with both positive symptoms and aggression in schizophrenia via the ENIGMA consortium. Psychol Med. (2020) 50:2034–45. doi: 10.1017/S0033291719002149 PMC13196894 31615588

[33] Wang YM Zhang YY Wang Y Cao Q Zhang M . Task-related brain activation associated with violence in patients with schizophrenia: A meta-analysis. Asian J Psychiatr. (2024) 97:104080. doi: 10.1016/j.ajp.2024.104080 38788320

[34] Singh JP Volavka J Czobor P Van Dorn RA . A meta-analysis of the Val158Met COMT polymorphism and violent behavior in schizophrenia. PLoS One. (2012) 7:e43423. doi: 10.1371/journal.pone.0043423 22905266 PMC3419214

[35] Bhakta SG Zhang JP Malhotra AK . The COMT Met158 allele and violence in schizophrenia: a meta-analysis. Schizophr Res. (2012) 140:192–7. doi: 10.1016/j.schres.2012.06.026 PMC4412346 22784685

[36] Lee PH Doyle AE Silberstein M Jung JY Liu RT Perlis RH . Associations between genetic risk for adult suicide attempt and suicidal behaviors in young children in the US. JAMA Psychiatry. (2022) 79:971–80. doi: 10.1001/jamapsychiatry.2022.2379 PMC9434482 36044238

[37] Xiang C Liu S Fan Y Wang X Jia Y Li L . Single nucleotide polymorphisms, variable number tandem repeats and allele influence on serotonergic enzyme modulators for aggressive and suicidal behaviors: A review. Pharmacol Biochem Behav. (2019) 180:74–82. doi: 10.1016/j.pbb.2019.03.008 30928299

[38] Koyama E Zai CC Bryushkova L Kennedy JL Beitchman JH . Predicting risk of suicidal ideation in youth using a multigene panel for impulsive aggression. Psychiatry Res. (2020) 285:112726. doi: 10.1016/j.psychres.2019.112726 31870620

[39] Reich R Gilbert A Clari R Burdick KE Szeszko PR . A preliminary investigation of impulsivity, aggression and white matter in patients with bipolar disorder and a suicide attempt history. J Affect Disord. (2019) 247:88–96. doi: 10.1016/j.jad.2019.01.001 30658245

[40] Lippard ETC Johnston JAY Spencer L Quatrano S Fan S Sankar A . Preliminary examination of gray and white matter structure and longitudinal structural changes in frontal systems associated with future suicide attempts in adolescents and young adults with mood disorders. J Affect Disord. (2019) 245:1139–48. doi: 10.1016/j.jad.2018.11.097 PMC6487887 30699858

[41] Drachman R Colic L Sankar A Spencer L Goldman DA Villa LM . Rethinking “aggression” and impulsivity in bipolar disorder: Risk, clinical and brain circuitry features. J Affect Disord. (2022) 303:331–9. doi: 10.1016/j.jad.2022.02.047 PMC9109470 35181384

[42] Lee Y Gilbert JR Waldman LR Zarate CA Jr Ballard ED . Potential association between suicide risk, aggression, impulsivity, and the somatosensory system. Soc Cognit Affect Neurosci. (2024) 19(1):nsae041. doi: 10.1093/scan/nsae041 38874947 PMC11219302

[43] Popova NK Tsybko AS Naumenko VS . The implication of 5-HT receptor family members in aggression, depression and suicide: similarity and difference. Int J Mol Sci. (2022) 23:8814. doi: 10.3390/ijms23158814 35955946 PMC9369404

[44] Anguelova M Benkelfat C Turecki G . A systematic review of association studies investigating genes coding for serotonin receptors and the serotonin transporter: II. Suicidal behavior. Mol Psychiatry. (2003) 8:646–53. doi: 10.1038/sj.mp.4001336 12874600

[45] Antypa N Serretti A Rujescu D . Serotonergic genes and suicide: a systematic review. Eur Neuropsychopharmacol. (2013) 23:1125–42. doi: 10.1016/j.euroneuro.2013.03.013 23742855

[46] Volavka J Citrome L . Pathways to aggression in schizophrenia affect results of treatment. Schizophr Bull. (2011) 37:921–9. doi: 10.1093/schbul/sbr041 PMC3160235 21562140

[47] Elyadini B Chakit M Elkhatir A Fitah I Khadmaoui A . Psychological assessment of violent behaviors in schizophrenic patients followed up in My EL Hassan health center of Kenitra, Morocco. Middle East Curr Psychiatr. (2024) 31:67. doi: 10.1186/s43045-024-00456-z

[48] Giegling I Olgiati P Hartmann AM Calati R Möller HJ Rujescu D . Personality and attempted suicide. Analysis of anger, aggression and impulsivity. J Psychiatr Res. (2009) 43:1262–71. doi: 10.1016/j.jpsychires.2009.04.013 19481222

[49] Albayrak Y Ekinci O Cayköylü A . Temperament and character personality profile in relation to suicide attempts in patients with schizophrenia. Compr Psychiatry. (2012) 53:1130–6. doi: 10.1016/j.comppsych.2012.04.007 22682677

[50] Canal-Rivero M Ayesa-Arriola R Setién-Suero E Crespo-Facorro B Arango C Dutta R . Understanding the influence of personality traits on risk of suicidal behaviour in schizophrenia spectrum disorders: A systematic review. J Clin Med. (2021) 10:b2535. doi: 10.3390/jcm10194604 PMC8509679 34640622

[51] Moher D Liberati A Tetzlaff J Altman DG PRISMA Group . Preferred reporting items for systematic reviews and meta-analyses: the PRISMA statement. BMJ. (2009) 339:b2535. doi: 10.1136/bmj.b2535 19622551 PMC2714657

[52] Higgins JPT Morgan RL Rooney AA Taylor KW Thayer KA Silva RA . A tool to assess risk of bias in non-randomized follow-up studies of exposure effects (ROBINS-E). Environ Int. (2024) 186:108602. doi: 10.1016/j.envint.2024.108602 38555664 PMC11098530

[53] McGuinness LA Higgins JPT . Risk-of-bias VISualization (robvis): An R package and Shiny web app for visualizing risk-of-bias assessments. Res Synth Methods. (2021) 12:55–61. doi: 10.1002/jrsm.1411 32336025

[54] Lin Y Wei Q Luo S Ye X Zheng F Zheng L . The risk of suicidal intention in severe mental illness: An ecological perspective. Psychol Psychother. (2023) 96:1044–61. doi: 10.1111/papt.12495 37668301

[55] Mork E Walby FA Harkavy-Friedman JM Barrett EA Steen NE Lorentzen S . Clinical characteristics in schizophrenia patients with or without suicide attempts and non-suicidal self-harm–a cross-sectional study. BMC Psychiatry. (2013) 13:255. doi: 10.1186/1471-244X-13-255 24106884 PMC3852098

[56] Köşger F Eşsizoğlu A Sönmez İ Güleç G Genek M Akarsu Ö . The relationship between violence and clinical features, insight and cognitive functions in patients with schizophrenia. Turk Psikiyatri Derg. (2016) 27:0. 27370060

[57] Iancu I Bodner E Roitman S Piccone Sapir A Poreh A Kotler M . Impulsivity, aggression and suicide risk among male schizophrenia patients. Psychopathology. (2010) 43:223–9. doi: 10.1159/000313520 20424503

[58] Lejoyeux M Nivoli F Basquin A Petit A Chalvin F Embouazza H . An investigation of factors increasing the risk of aggressive behavior among schizophrenic inpatients. Front Psychiatry. (2013) 4:97. doi: 10.3389/fpsyt.2013.00097 24027539 PMC3759799

[59] Tousignant M Pouliot L Routhier D Vrakas G McGirr A Turecki G . Suicide, schizophrenia, and schizoid-type psychosis: role of life events and childhood factors. Suicide Life Threat Behav. (2011) 41:66–78. doi: 10.1111/j.1943-278X.2010.00002.x 21309825

[60] Neuner T Hübner-Liebermann B Hausner H Hajak G Wolfersdorf M Spiessl H . Revisiting the association of aggression and suicidal behavior in schizophrenic inpatients. Suicide Life Threat Behav. (2011) 41:171–9. doi: 10.1111/j.1943-278X.2011.00018.x 21342219

[61] Yıldız M Yazıcı A Böke O . Demographic and clinical characteristics in schizophrenia: a multi center cross-sectional case record study. Turk Psikiyatri Derg. (2010) 21:213–24. 20818509

[62] Hoaken PNS Stewart SH . Drugs of abuse and the elicitation of human aggressive behavior. Addict Behav. (2003) 28:1533–54. doi: 10.1016/j.addbeh.2003.08.033 14656544

[63] Lamsma J Cahn W Fazel S GROUP and NEDEN investigators . Use of illicit substances and violent behaviour in psychotic disorders: two nationwide case-control studies and meta-analyses. Psychol Med. (2020) 50:2028–33. doi: 10.1017/S0033291719002125 PMC7525769 31462346

[64] Buckley PF Miller BJ Lehrer DS Castle DJ . Psychiatric comorbidities and schizophrenia. Schizophr Bull. (2009) 35:383–402. doi: 10.1093/schbul/sbn135 19011234 PMC2659306

[65] Li Y Li Y Cao J . Factors associated with suicidal behaviors in mainland China: a meta-analysis. BMC Public Health. (2012) 12:524. doi: 10.1186/1471-2458-12-524 22800121 PMC3490836

[66] Hawton K Casañas I Comabella C Haw C Saunders K . Risk factors for suicide in individuals with depression: A systematic review. J Affect Disord. (2013) 147:17–28. doi: 10.1016/j.jad.2013.01.004 23411024

[67] McGirr A Turecki G . What is specific to suicide in schizophrenia disorder? Demographic, clinical and behavioural dimensions. Schizophr Res. (2008) 98:217–24. doi: 10.1016/j.schres.2007.09.009 17942279

[68] Chong BTW Wahab S Muthukrishnan A Tan KL Ch’ng ML Yoong MT . Prevalence and factors associated with suicidal ideation in institutionalized patients with schizophrenia. Psychol Res Behav Manag. (2020) 13:949–62. doi: 10.2147/PRBM.S266976 PMC7667143 33204188

[69] Liu J Zhao K Zhou S Hong L Xu Y Sun S . Suicidal ideation in Chinese adults with schizophrenia: associations with neurocognitive function and empathy. BMC Psychiatry. (2023) 23:311. doi: 10.1186/s12888-023-04739-3 37138258 PMC10155378

[70] Dumais A Lesage AD Alda M Rouleau G Dumont M Chawky N . Risk factors for suicide completion in major depression: a case-control study of impulsive and aggressive behaviors in men. Am J Psychiatry. (2005) 162:2116–24. doi: 10.1176/appi.ajp.162.11.2116 16263852

[71] Pompili M Innamorati M Raja M Falcone I Ducci G Angeletti G . Suicide risk in depression and bipolar disorder: Do impulsiveness-aggressiveness and pharmacotherapy predict suicidal intent? Neuropsychiatr Dis Treat. (2008) 4:247–55. doi: 10.2147/ndt.s2192 PMC2515901 18728807

[72] Scholte-Stalenhoef AN Pijnenborg GHM Hasson-Ohayon I Boyette LL . Personality traits in psychotic illness and their clinical correlates: A systematic review. Schizophr Res. (2023) 252:348–406. doi: 10.1016/j.schres.2023.01.001 36804473

[73] de Jong JJ de Gelder B Hodiamont PPPG . Sensory processing, neurocognition, and social cognition in schizophrenia: towards a cohesive cognitive model. Schizophr Res. (2013) 146:209–16. doi: 10.1016/j.schres.2013.02.034 23522906

[74] Tripathi A Kar SK Shukla R . Cognitive deficits in schizophrenia: understanding the biological correlates and remediation strategies. Clin Psychopharmacol Neurosci. (2018) 16:7–17. doi: 10.9758/cpn.2018.16.1.7 29397662 PMC5810454

[75] Khazaie H Hamzeh B Najafi F Chehri A Rahimi-Movaghar A Amin-Esmaeili M . Co-occurrence of aggression and suicide attempt among young people and related factors: findings from Iranian youth cohort study in ravansar. Arch Iran Med. (2023) 26:322–9. doi: 10.34172/aim.2023.49 PMC10685835 38310433

[76] Arunpongpaisal S Assanangkornchai S Chongsuvivatwong V . Developing a risk prediction model for death at first suicide attempt-Identifying risk factors from Thailand’s national suicide surveillance system data. PLoS One. (2024) 19:e0297904. doi: 10.1371/journal.pone.0297904 38598456 PMC11006158

[77] O’Hare K Fadiloglu K Lång U Healy C Cannon M DeVylder J . Psychotic experiences and risk of suicidal thoughts and behaviors: A systematic review and meta-analysis of longitudinal population studies. Schizophr Bull. (2024) 16:sbae197. doi: 10.1093/schbul/sbae197 PMC12809804 39550208

[78] Bolton JM Belik SL Enns MW Cox BJ Sareen J . Exploring the correlates of suicide attempts among individuals with major depressive disorder: findings from the national epidemiologic survey on alcohol and related conditions. J Clin Psychiatry. (2008) 69:1139–49. doi: 10.4088/jcp.v69n0714 18517287

[79] Le GH Wong S Au H Badulescu S Gill H Vasudeva S . Association between rumination, suicidal ideation and suicide attempts in persons with depressive and other mood disorders and healthy controls: A systematic review and meta-analysis. J Affect Disord. (2025) 368:513–27. doi: 10.1016/j.jad.2024.09.118 39303880

[80] Mash HBH Ursano RJ Kessler RC Naifeh JA Fullerton CS Aliaga PA . Predictors of suicide attempt within 30 days of first medically documented major depression diagnosis in U.S. army soldiers with no prior suicidal ideation. BMC Psychiatry. (2023) 23:392. doi: 10.1186/s12888-023-04872-z 37268952 PMC10239190

[81] Cong X Zhang T Bian R Li Y Liu J Zhang X . Prevalence and related factors of first-time suicide attempts in the past 14 days in Chinese adult patients with first-episode drug-naïve major depressive disorder. Front Psychiatry. (2024) 15:1366475. doi: 10.3389/fpsyt.2024.1366475 38585486 PMC10995384

[82] Amanollahi M Jameie M Looha MA A Basti F Cattarinussi G Moghaddam HS . Machine learning applied to the prediction of relapse, hospitalization, and suicide in bipolar disorder using neuroimaging and clinical data: A systematic review. J Affect Disord. (2024) 361:778–97. doi: 10.1016/j.jad.2024.06.061 38908556

[83] Walsh CG Johnson KB Ripperger M Sperry S Harris J Clark N . Prospective validation of an electronic health record-based, real-time suicide risk model. JAMA Netw Open. (2021) 4:e211428. doi: 10.1001/jamanetworkopen.2021.1428 33710291 PMC7955273

[84] Haroz EE Rebman P Goklish N Garcia M Suttle R Maggio D . Performance of machine learning suicide risk models in an American Indian population. JAMA Netw Open. (2024) 7:e2439269. doi: 10.1001/jamanetworkopen.2024.39269 39401036 PMC11474420

[85] Ehtemam H Sadeghi Esfahlani S Sanaei A Ghaemi MM Hajesmaeel-Gohari S Rahimisadegh R . Role of machine learning algorithms in suicide risk prediction: a systematic review-meta analysis of clinical studies. BMC Med Inform Decis Mak. (2024) 24:138. doi: 10.1186/s12911-024-02524-0 38802823 PMC11129374

[86] Corke M Mullin K Angel-Scott H Xia S Large M . Meta-analysis of the strength of exploratory suicide prediction models; from clinicians to computers. BJPsych Open. (2021) 7:e26. doi: 10.1192/bjo.2020.162 33407984 PMC8058929

[87] Somé NH Noormohammadpour P Lange S . The use of machine learning on administrative and survey data to predict suicidal thoughts and behaviors: a systematic review. Front Psychiatry. (2024) 15:1291362. doi: 10.3389/fpsyt.2024.1291362 38501090 PMC10944962

[88] Fazel S Wolf A Larsson H Mallett S Fanshawe TR . The prediction of suicide in severe mental illness: development and validation of a clinical prediction rule (OxMIS). Transl Psychiatry. (2019) 9:98. doi: 10.1038/s41398-019-0428-3 30804323 PMC6389890

[89] Sariaslan A Fanshawe T Pitkänen J Cipriani A Martikainen P Fazel S . Predicting suicide risk in 137,112 people with severe mental illness in Finland: external validation of the Oxford Mental Illness and Suicide tool (OxMIS). Transl Psychiatry. (2023) 13:126. doi: 10.1038/s41398-023-02422-5 37072392 PMC10113231

[90] Kim SW Kim SJ Mun JW Bae KY Kim JM Kim SY . Psychosocial factors contributing to suicidal ideation in hospitalized schizophrenia patients in Korea. Psychiatry Invest. (2010) 7:79–85. doi: 10.4306/pi.2010.7.2.79 PMC2890872 20577615

[91] Bouhlel S M’solly M Benhawala S Jones Y El-Hechmi Z . Factors related to suicide attempts in a Tunisian sample of patients with schizophrenia. Encephale. (2013) 39:6–12. doi: 10.1016/j.encep.2012.06.003 23095582

[92] Woottiluk P Maneeton B Jaiyen N Khemawichanurat W Kawilapat S Maneeton N . Prevalence and associated factors of suicide among hospitalized schizophrenic patients. World J Clin cases. (2020) 8:757–70. doi: 10.12998/wjcc.v8.i4.757 PMC7052544 32149059

[93] Probert-Lindström S Berge J Westrin Å Öjehagen A Skogman Pavulans K . Long-term risk factors for suicide in suicide attempters examined at a medical emergency in patient unit: results from a 32-year follow-up study. BMJ Open. (2020) 10:e038794. doi: 10.1136/bmjopen-2020-038794 PMC7783608 33130567

[94] Appleby L Shaw J Amos T McDonnell R Harris C McCann K . Suicide within 12 months of contact with mental health services: national clinical survey. BMJ. (1999) 318:1235–9. doi: 10.1136/bmj.318.7193.1235 PMC27859 10231250

[95] Olfson M Wall M Wang S Crystal S Liu SM Gerhard T . Short-term suicide risk after psychiatric hospital discharge. JAMA Psychiatry. (2016) 73:1119–26. doi: 10.1001/jamapsychiatry.2016.2035 PMC8259698 27654151

[96] Cha Y Links PS Ba D Kazi A . Systematic review of the effectiveness and experiences of treatment for men with borderline personality disorder. Am J Mens Health. (2024) 18:15579883241271894. doi: 10.1177/15579883241271894 39215612 PMC11367612

[97] Gaynes BN Brown CL Lux LJ Brownley KA Van Dorn RA Edlund MJ . Preventing and de-escalating aggressive behavior among adult psychiatric patients: A systematic review of the evidence. Psychiatr Serv. (2017) 68:819–31. doi: 10.1176/appi.ps.201600314 28412887

[98] Pompili M Lester D Dominici G Longo L Marconi G Forte A . Indications for electroconvulsive treatment in schizophrenia: a systematic review. Schizophr Res. (2013) 146:1–9. doi: 10.1016/j.schres.2013.02.005 23499244

[99] Hu FH Xu J Jia YJ Ge MW Zhang WQ Tang W . Non-pharmacological interventions for preventing suicide attempts: A systematic review and network meta-analysis. Asian J Psychiatr. (2024) 93:103913. doi: 10.1016/j.ajp.2024.103913 38219553

[100] Lecomte T Spidel A Leclerc C MacEwan GW Greaves C Bentall RP . Predictors and profiles of treatment non-adherence and engagement in services problems in early psychosis. Schizophr Res. (2008) 102:295–302. doi: 10.1016/j.schres.2008.01.024 18295458

[101] Czobor P Van Dorn RA Citrome L Kahn RS Fleischhacker WW Volavka J . Treatment adherence in schizophrenia: a patient-level meta-analysis of combined CATIE and EUFEST studies. Eur Neuropsychopharmacol. (2015) 25:1158–66. doi: 10.1016/j.euroneuro.2015.04.003 PMC4860611 26004980

[102] Smeijers D Bulten E Buitelaar J Verkes RJ . Treatment responsivity of aggressive forensic psychiatric outpatients. Int J Offender Ther Comp Criminol. (2018) 62:3834–52. doi: 10.1177/0306624X17747052 PMC6094550 29254396

[103] Bader D Frank K . Understanding experiences of non-physical maltreatment in childhood in Canada: What is the relationship with suicidal ideation and mental health disorders? Health Re. (2024) 35:16–28. doi: 10.25318/82-003-x202400900002-eng 39292857

[104] Oei A Li D Chu CM Ng I Hoo E Ruby K . Disruptive behaviors, antisocial attitudes, and aggression in young offenders: Comparison of Adverse Childhood Experience (ACE) typologies. Child Abuse Negl. (2023) 141:106191. doi: 10.1016/j.chiabu.2023.106191 37084615

[105] Koyama E Kant T Takata A Kennedy JL Zai CC . Genetics of child aggression, a systematic review. Transl Psychiatry. (2024) 14:252. doi: 10.1038/s41398-024-02870-7 38862490 PMC11167064

[106] van der Stouwe T Leijten P Asscher JJ Deković M van der Put CE . Adding structured components to home visitation to reduce mothers’ Risk for child maltreatment: a randomized controlled trial. J Fam Violence. (2023) 13:1–14. doi: 10.1007/s10896-023-00509-7 PMC9924864 36817847

[107] de Wit M Leijten P van der Put C Asscher J Bouwmeester-Landweer M Deković M . Study protocol: randomized controlled trial of manualized components in home visitation to reduce mothers’ risk for child maltreatment. BMC Public Health. (2020) 20:136. doi: 10.1186/s12889-020-8237-4 32000744 PMC6993430

[108] Jones KA Freijah I Brennan SE McKenzie JE Bright TM Fiolet R . Interventions from pregnancy to two years after birth for parents experiencing complex post-traumatic stress disorder and/or with childhood experience of maltreatment. Cochrane Database Syst Rev. (2023) 5:CD014874. doi: 10.1002/14651858.CD014874.pub2 37146219 PMC10162699

[109] Meerwijk EL Parekh A Oquendo MA Allen IE Franck LS Lee KA . Direct versus indirect psychosocial and behavioural interventions to prevent suicide and suicide attempts: a systematic review and meta-analysis. Lancet Psychiatry. (2016) 3:544–54. doi: 10.1016/S2215-0366(16)00064-X 27017086

[110] Nibbio G Bertoni L Calzavara-Pinton I Necchini N Paolini S Baglioni A . The relationship between cognitive impairment and violent behavior in people living with schizophrenia spectrum disorders: A critical review and treatment considerations. Medicina (Kaunas). (2024) 60(8):1261. doi: 10.3390/medicina60081261 39202542 PMC11356552

[111] Barlati S Nibbio G Stanga V Giovannoli G Calzavara-Pinton I Necchini N . Cognitive and clinical characteristics of offenders and non-offenders diagnosed with schizophrenia spectrum disorders: results of the Recoviwel observational study. Eur Arch Psychiatry Clin Neurosci. (2023) 273:1307–16. doi: 10.1007/s00406-022-01510-9 36309882

[112] Brokke SS Landrø NI Haaland VØ . Cognitive control in suicide ideators and suicide attempters. Front Psychol. (2020) 11:595673. doi: 10.3389/fpsyg.2020.595673 33424712 PMC7785752

[113] Jager-Hyman S Cunningham A Wenzel A Mattei S Brown GK Beck AT . Cognitive distortions and suicide attempts. Cognit Ther Res. (2014) 38:369–74. doi: 10.1007/s10608-014-9613-0 PMC4185206 25294949

[114] Lalovic A Wang S Keilp JG Bowie CR Kennedy SH Rizvi SJ . A qualitative systematic review of neurocognition in suicide ideators and attempters: Implications for cognitive-based psychotherapeutic interventions. Neurosci Biobehav Rev. (2022) 132:92–109. doi: 10.1016/j.neubiorev.2021.11.007 34774586

[115] Gebreegziabhere Y Habatmu K Mihretu A Cella M Alem A . Cognitive impairment in people with schizophrenia: an umbrella review. Eur Arch Psychiatry Clin Neurosci. (2022) 272:1139–55. doi: 10.1007/s00406-022-01416-6 PMC9508017 35633394

[116] Vita A Nibbio G Barlati S . Conceptualization and characterization of “primary” and “secondary” cognitive impairment in schizophrenia. Psychiatry Res. (2024) 340:116126. doi: 10.1016/j.psychres.2024.116126 39128169

[117] Bombassaro T Carrilho CG Peixoto C Alves GS Kahn JP Nardi AE . Cognition in schizophrenia: A systematic review of wechsler adult intelligence scale studies. Prim Care Companion CNS Disord. (2023) 25(5):22r03456. doi: 10.4088/PCC.22r03456 37857289

[118] Vita A Gaebel W Mucci A Sachs G Barlati S Giordano GM . European Psychiatric Association guidance on treatment of cognitive impairment in schizophrenia. Eur Psychiatry. (2022) 65:e57. doi: 10.1192/j.eurpsy.2022.2315 36059103 PMC9532218

[119] Machetanz L Hofmann AB Möhrke J Kirchebner J . Offenders and non-offenders with schizophrenia spectrum disorders: the crime-preventive potential of sufficient embedment in the mental healthcare and support system. Front Psychiatry. (2023) 14:1231851. doi: 10.3389/fpsyt.2023.1231851 37711423 PMC10498463

[120] Lejeune JA Northrop A Kurtz MM . A meta-analysis of cognitive remediation for schizophrenia: efficacy and the role of participant and treatment factors. Schizophr Bull. (2021) 47:997–1006. doi: 10.1093/schbul/sbab022 33772310 PMC8266668

[121] Shimada T Ito S Makabe A Yamanushi A Takenaka A Kawano K . Aerobic exercise and cognitive functioning in schizophrenia: An updated systematic review and meta-analysis. Psychiatry Res. (2022) 314:114656. doi: 10.1016/j.psychres.2022.114656 35659670

[122] Calzavara-Pinton I Nibbio G Barlati S Bertoni L Necchini N Zardini D . Treatment of cognitive impairment associated with schizophrenia spectrum disorders: new evidence, challenges, and future perspectives. Brain Sci. (2024) 14(8):791. doi: 10.3390/brainsci14080791 39199483 PMC11352256

[123] Hemager N Plessen KJ Thorup A Christiani C Ellersgaard D Spang KS . Assessment of neurocognitive functions in 7-year-old children at familial high risk for schizophrenia or bipolar disorder: the danish high risk and resilience study VIA 7. JAMA Psychiatry. (2018) 75:844–52. doi: 10.1001/jamapsychiatry.2018.1415 PMC6143091 29926086

[124] Davies G Marioni RE Liewald DC Hill WD Hagenaars SP Harris SE . Genome-wide association study of cognitive functions and educational attainment in UK Biobank (N=112 151). Mol Psychiatry. (2016) 21:758–67. doi: 10.1038/mp.2016.45 PMC4879186 27046643

[125] Aas M Dazzan P Mondelli V Melle I Murray RM Pariante CM . A systematic review of cognitive function in first-episode psychosis, including a discussion on childhood trauma, stress, and inflammation. Front Psychiatry. (2014) 4:182. doi: 10.3389/fpsyt.2013.00182 24409157 PMC3884147

[126] Aguirre JM Díaz Dellarossa C Barbagelata D Vásquez J Mena C Tepper Á . Cognitive function at first episode in patients subsequently developing treatment-resistant schizophrenia. Schizophr Res. (2025) 276:178–84. doi: 10.1016/j.schres.2025.01.017 39893777

[127] Bora E Murray RM . Meta-analysis of cognitive deficits in ultra-high risk to psychosis and first-episode psychosis: do the cognitive deficits progress over, or after, the onset of psychosis? Schizophr Bull. (2014) 40:744–55. doi: 10.1093/schbul/sbt085 PMC4059428 23770934

[128] Hsiung SC Adlersberg M Arango V Mann JJ Tamir H Liu KP . Attenuated 5-HT1A receptor signaling in brains of suicide victims: involvement of adenylyl cyclase, phosphatidylinositol 3-kinase, Akt and mitogen-activated protein kinase. J Neurochem. (2003) 87:182–94. doi: 10.1046/j.1471-4159.2003.01987.x 12969265

[129] Fitzgerald ML Kassir SA Underwood MD Bakalian MJ Mann JJ Arango V . Dysregulation of striatal dopamine receptor binding in suicide. Neuropsychopharmacology. (2017) 42:974–82. doi: 10.1038/npp.2016.124 PMC5312055 27402414

[130] Zhang L Verwer RWH Lucassen PJ Huitinga I Swaab DF . Prefrontal cortex alterations in glia gene expression in schizophrenia with and without suicide. J Psychiatr Res. (2020) 121:31–8. doi: 10.1016/j.jpsychires.2019.11.002 31739114

[131] Weltens I Bak M Verhagen S Vandenberk E Domen P van Amelsvoort T . Aggression on the psychiatric ward: Prevalence and risk factors. A systematic review of the literature. PLoS One. (2021) 16:e0258346. doi: 10.1371/journal.pone.0258346 34624057 PMC8500453

[132] Laitano HV Ely A Sordi AO Schuch FB Pechansky F Hartmann T . Anger and substance abuse: a systematic review and meta-analysis. Braz J Psychiatry. (2022) 44:103–10. doi: 10.1590/1516-4446-2020-1133 PMC8827371 33605366

[133] Shima C Lee R Coccaro EF . Associations of agression and use of caffeine, alcohol and nicotine in healthy and aggressive individuals. J Psychiatr Res. (2022) 146:21–7. doi: 10.1016/j.jpsychires.2021.10.015 34942448

